# Real-time Kinematic Positioning of INS Tightly Aided Multi-GNSS Ionospheric Constrained PPP

**DOI:** 10.1038/srep30488

**Published:** 2016-07-29

**Authors:** Zhouzheng Gao, Wenbin Shen, Hongping Zhang, Xiaoji Niu, Maorong Ge

**Affiliations:** 1 School of Geodesy and Geomatics, Wuhan University, 129 Luoyu Road, Wuhan 430079, China; 2 GNSS Research Center, Wuhan University, 129 Luoyu Road, Wuhan 430079, China; 3 German Research Centre for Geosciences (GFZ), Telegrafenberg, Potsdam 14473, Germany

## Abstract

Real-time Precise Point Positioning (PPP) technique is being widely applied for providing precise positioning services with the significant improvement on satellite precise products accuracy. With the rapid development of the multi-constellation Global Navigation Satellite Systems (multi-GNSS), currently, about 80 navigation satellites are operational in orbit. Obviously, PPP performance is dramatically improved with all satellites compared to that of GPS-only PPP. However, the performance of PPP could be evidently affected by unexpected and unavoidable severe observing environments, especially in the dynamic applications. Consequently, we apply Inertial Navigation System (INS) to the Ionospheric-Constrained (IC) PPP to overcome such drawbacks. The INS tightly aided multi-GNSS IC-PPP model can make full use of GNSS and INS observations to improve the PPP performance in terms of accuracy, availability, continuity, and convergence speed. Then, a set of airborne data is analyzed to evaluate and validate the improvement of multi-GNSS and INS on the performance of IC-PPP.

In last decades, the Global Navigation Satellite System (GNSS) has been developed dramatically and is applied widely in scientific researches and engineering applications^1^,^2^,^3^,^4^,^5^,^6^,^7^. For precise positioning and navigation, there are two major techniques, i.e. the Precise Point Positioning (PPP)^8^ and the Real Time Kinematic (RTK)^9^,^10^ techniques. Although RTK can provide positioning of high accuracy with rapid convergence in both real-time and post mode, it needs dedicated base stations and its performance degraded visibly along with the distance between base station and users. These weaknesses limit the applications of RTK in the regions where no nearby base stations are available, such as in large area aerial surveying, marine surveying, and positioning in outlying areas. Fortunately, due to the significant improvement on accuracy for satellite orbit and clock products^11^, PPP technology becomes a potential precise positioning usage in nowadays, especially after the Real-time Service (RTS) project of International GNSS Service (IGS) became operational. However, the accuracy, availability, continuity, and the convergence time of GNSS PPP will still be influenced by satellite availability, spatial geometrical strength between satellites and users measured by Position Dilution of Precision (PDOP), observation quality, Un-calibrated Phase Delay (UPD) etc.^12^,^13^,^14^, especially for dynamic applications. More recently, several research works have been carried out such as the ambiguity resolution^13^,^14^, ionospheric constrained PPP^15^,^16^, GLONASS aided GPS PPP^17^,^18^ in order to enhance the performance of PPP. Thanks to the rapid development of GNSS constellations and Inertial Navigation System (INS) technology, the multi-constellation GNSS^17^,^18^,^19^,^20^ and INS^21^,^22^,^23^ are being paid more attention to enhance the performance of PPP.

Currently, the American GPS and the Russia GLONASS with full constellation of thirty-two and twenty-four Medium Earth Orbit (MEO) satellites, respectively, provide global coverage and have got the Full Operational Capability (FOC) for all users^19^,^20^,^24^. Besides, there are two emerging GNSS constellations namely Galileo developed by European Union (EU) and BeiDou developed by China^24^. At present, the Galileo finished its In Orbit Validation (IOV) phase with the constellation of four IOV satellites and followed by launching eight full operational capability satellites in the Medium Earth Orbit^24^. According to its plane, the full constellation of Galileo will be consist of thirty satellites in three orbit planes and will provide more accurate Positioning, Navigation, and Timing (PNT) services for all users free. The BeiDou navigation satellite system (BDS) has been developed independently by China since 2011, and it has obtained its initial operational capability for the Asian-Pacific region at the end of 2012 with the constellation of five Geostationary Earth Orbit (GEO) satellites, five Inclined Geo-Synchronous Orbit (IGSO) satellites, and four MEO satellites^24^,^25^. Another five BeiDou satellites with Inter-satellite links (ISL) function were launched in 2015. By the end of 2020, the BDS will complete full operational capability with five GEOs, three IGSOs, and twenty-seven MEOs. Significantly, up to now, there are more than eighty GNSS satellites working in the orbit and more than twenty satellites can be used in PNT service at the same time in open sky condition compared to thirty-two GPS satellites in view and about fourteen or fewer GPS satellites available at each epoch. According to previous studies, the satellite availability, spatial geometrical strength, and the corresponding PDOP can be improved evidently once multi-GNSS data are used together^26^, which will also enhance visibly the PPP performance such as accuracy, availability, and continuity^19^,^20^.

However, both GPS and multi-GNSS cannot work well in complex environments e.g. city urban, overpass, umbrage, and tunnel etc. Because such surroundings can cause partial or full GNSS signal outages frequently, which will result in re-initializations, low accuracy, and discontinuity even untrustworthy for GNSS solutions. To improve the performance of GNSS in such environment, an effective way is to combine GNSS with INS to form a new integrated system which can utilize the advantages (e.g. GNSS high positioning accuracy and INS automatic positioning characters) of each individual system^21^,^22^,^23^,^27^,^28^,^29^. What is more, the new system can overcome the disadvantages of the two systems such that INS accuracy is influenced by IMU sensor errors significantly along with the increasing time^30^,^31^,^32^ and the poor performance of GNSS in severe dynamic conditions. According to the previous studies on the INS tightly aided GPS PPP, the integrated system can not only improve PPP performance, aid the recovery of GNSS data gaps^22^, and enhance the re-initialization evidently^23^, but also decrease the position drifts of caused by IMU sensors’ hardware errors^21^,^22^,^23^,^29^.

Currently, all of the previous studies about INS aided PPP are based on single- and multi-GNSS ionosphere free combination^21^,^22^,^29^,^33^ or single-GNSS (GPS) Ionospheric Constrained (IC) PPP^23^. In the present study, we will introduce the new algorithm of INS tightly aided multi-GNSS (GPS + BeiDou + GLONASS) PPP using the GNSS raw observations with the ionosphere delays and receiver Differential Code Dias (DCB) constrained. Its significant contribution to PPP compared to current INS aided GPS PPP will be demonstrated. The mathematic model of the INS tightly aided multi-GNSS IC-PPP is described in detail in the “Method” part. Succinctly, the raw pseudo-range and carrier-phase observations instead of the ionospheric-free combination values are used in the IC-PPP model. Meanwhile, the Inter System Bias (ISB) and Inter Frequency Bias (IFB) as well as the DCB of pseudo-range of GNSS receiver and the ionosphere delays in line of sight on one frequency are estimated as parameters in a unique estimator. In order to assess the performance of the new system, a set of airborne data is processed and analyzed in the “Results” part. The impacts of availability and PDOP of multi-GNSS and INS on the positioning accuracy, convergence time, and attitude determination will be assessed.

## Method

In the INS tightly aided IC-PPP model, the GNSS raw observations (pseudo-range, carrier-phase, and Doppler) and the INS solutions (positions, velocities, and attitudes) are combined in one filter^22^,^23^. Simply, the observation model can be expressed as


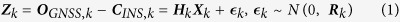


where ***Z***_*k*_ is the innovation vector at epoch *k* calculated by making difference between GNSS raw observations (***O***_*GNSS*_) and INS predicted GNSS values (***C***_*INS*_); ***H***_*k*_ denotes the designed matrix of the state parameter vector (***X***_*k*_); 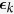 represents the observation noise with the apriori covariance ***R***_*k*_.

Generally, ***Z***_*k*_ for INS aided IC-PPP consists of the innovations of pseudo-range (***P***), carrier-phase (***L***), and Doppler (***D***) from two frequencies (*f* = 1,2), and the other virtual observations including ionospheric delays (***I***), zenith tropospheric delay (*T*), and receiver DCBs (***d***), which can be written as follows


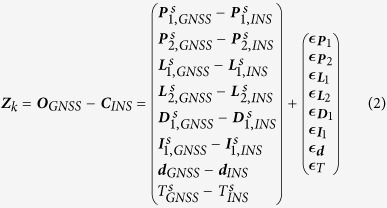


where *s* stands for the GNSS systems (*i.e*., GPS, GLONASS, and BeiDou); “*GNSS*” and “*INS*” indicate the GNSS raw observations and INS predicted values. For GPS + GLONASS + BeiDou three system data, the innovation functions for pseudo-range on frequency *f*_1_ and *f*_2_ (GPS: *L*_1_ and *L*_2_; BeiDou: *B*_1_ and *B*_2_; GLONASS: *L*_1,*m*_ and *L*_2,*m*_, *m* is the frequency number) can be further defined as


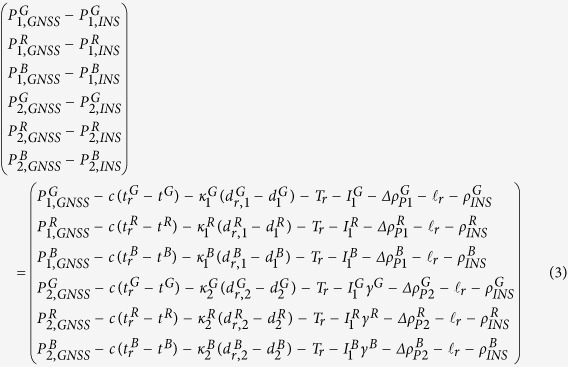


where *c* is the velocity of light in vacuum and the subscript *r* denotes GNSS receiver; the indices *G, R*, and *B* indicate GPS, GLONASS, and BeiDou respectively; *ρ*_*INS*_ is the geometry distance calculated by satellite positions and INS updated GNSS receiver position, here the receiver positions are provided by INS mechanization^32^ using the velocity increments from accelerometers after the error compensation; *t*_*r*_ and *t*^*s*^ represent receiver clock offset at the signal receiving time and satellite clock offset at the signal transmitting time; 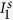 and *T*_*r*_ are ionospheric delay on *f*_1_ frequency and the tropospheric delay along the signal propagation path; 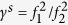 denotes the ionospheric delay on *f*_2_ by using ionospheric delay on *f*_1_; *κ* is the coefficient to transform DCB corrections to pseudo-ranges on *f*_1_ and *f*_2_, *i.e*., 
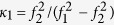
, 
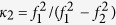
; 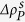 stands for other corrections in pseudo-range such as relativity effect, earth rotation effect, antenna Phase Center Offset (PCO) and Variation (PCV) of GNSS receiver and satellite etc.^11^, all of these errors will be corrected by the classic models^11^; 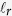 denotes the position lever arm correction between GNSS receiver phase center and IMU center; 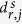 and 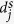 are DCB^14^,^15^,^16^,^23^ for GNSS receiver and satellite on frequency *j*.

Analogously, the innovation functions for carrier-phase on frequency *f*_1_ and *f*_2_ can be expressed as


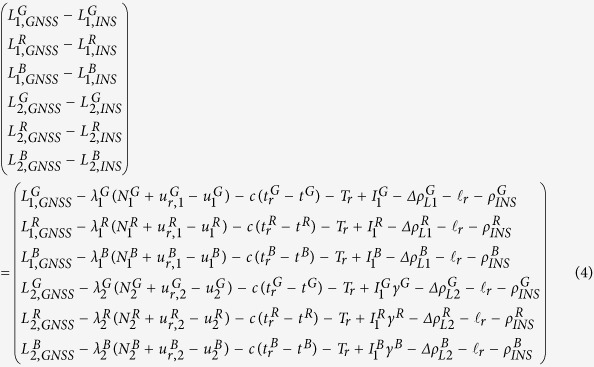


where *λ* and *N* represent carrier-phase wavelength and integer ambiguity, respectively; 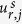 and 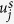 are un-calibrated phase delay^14^ of receiver and satellite on *j* frequency which can be absorbed by float ambiguity (
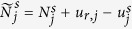
) in PPP parameter estimation, and the random constant model is used to describe the float ambiguities when no cycle slip happens; 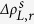 stands for other corrections in carrier-phase.

In this paper, the ionospheric delays at *f*_1_ frequency (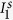) in line of sight is estimated as parameters^15^,^16^ instead of ionosphere-free combination adopted by convention PPP to eliminate the first order ionospheric delay due to its frequency dependent character, and the ionospheric delays at *f*_2_ frequency can be expressed as 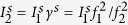. The ionospheric delay can be modeled as


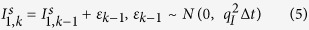


where 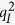 is the Power Spectral Density (PSD) of the ionospheric dynamic noise (*ε*_*k*−1_); Δ*t* is the interval between two adjacent epochs.

Since there are too many parameters that should be estimated in IC-PPP mode and there are high correlation between the ionospheric delays and the DCB^16^, the PPP solutions are rather weak. To enhance IC-PPP solution, Global Ionosphere Mapping (GIM) data provided by IGS are adopted as virtual observations to constrain the ionospheric delay for each satellite-user pair. The mathematic model can be expressed as^16^:


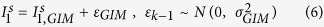


where 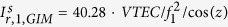 is the ionospheric delay calculated by GIM model with a priori variance 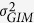 for its noise (*ε*_*GIM*_); *VTEC* and *z* denote vertical total electron content and zenith angle at ionosphere pierce point (IPP). Considering the temporal and spatial characteristics of ionospheric delay, 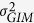 can be further written as


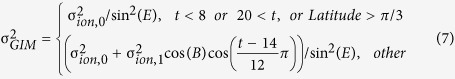


where *E* and *Latitude* indicate the satellite elevation angle and the latitude of the IPP; *t* is the local time at the IPP; 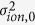 and 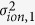 are the variance of the ionospheric zenith delay and the variance of ionospheric delay variation along with latitude and local time.

As a result, the innovation function for ionospheric delays can be written as


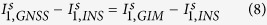


where 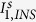 is a predicted value in INS aided IC-PPP.

Besides, according to the previous research about PPP, the DCBs can be absorbed by receiver clock and satellite clock in convention PPP mode^16^. However, such DCBs will not be absorbed by the single clock in IC-PPP mode, which will impact obviously on the initial position accuracy and the convergence time of IC-PPP^16^. Therefore, the satellite DCBs are eliminated using International GNSS Service (IGS) DCB products and the receiver DCB is estimated as parameter by modeling as random walk process in IC-PPP mode. The model for receiver DCB can be expressed as


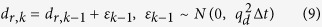


where 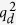 is the PSD of the DCB dynamic noise (*ε*_*k*−1_). And its innovation function can be expressed as ***d***_*GNSS*_ − ***d***_*INS*_ = 0.

The tropospheric delay consists of dry component and wet component, and both of the two can be expressed as a product of Zenith Tropospheric Delay (ZTD) and mapping function^34^. The dry delay can be modelled accurately and can be corrected precisely by classical model, but the wet one cannot be corrected well. To weaken the effect of the residual of the wet ZTD on IC-PPP performance, it can be modeled as a random process and estimated as parameter in IC-PPP, expressed as


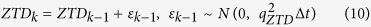


where 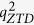 is the PSD of the wet ZTD dynamic noise (*ε*_*k*−1_). And the innovation function is defined as 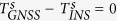.

The Doppler data can accelerate IMU sensor errors estimation and provide velocity solution for INS tightly aided IC-PPP. The innovation functions for Doppler at frequency *f*_1_ can be written as


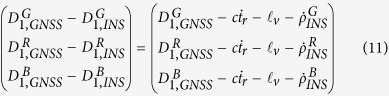


where 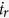, 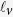, and 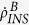 are receiver clock drift, velocity lever-arm correction, and the rate of distance change along the line of sight, respectively. Here, 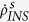 is computed by satellite velocities and INS predicted receiver velocities which are updated by INS mechanization^32^.

According to the mathematical functions above, the designed matrix ***H***_*k*_ can be obtained by differential of eqs (3,4 and 11). The final form of state parameter vector ***X***_*k*_ for the INS tightly aided IC-PPP can be written as


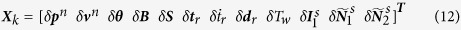


where *δ**p***^*n*^, *δ**v***^*n*^, and *δ**θ*** are the correction vectors of position, velocity, and attitude, respectively; *n* denotes local frame (North-East-Down); *δ**B*** and *δ**S*** are the correction vectors of biases and scale factors of IMU sensors; 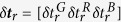,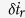, and 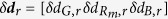 stand for the receiver clock offset correction vector, clock drift correction, and receiver DCBs correction, respectively; *δT*_*w*_ and 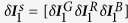 indicate the residual of the wet component of the tropospheric zenith delay and ionospheric delays correction; 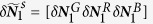
and 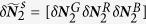 represent the float ambiguity corrections.

Then, the extend Kalmam filter (EKF) is utilized for parameters estimation, and the corresponding state function can be expressed as


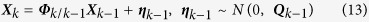


where ***Φ***_*k*/*k*−1_ is the state transfer matrix from epoch *k*-1 to epoch *k*, which can be derived by state parameter dynamic functions; ***η*** denotes state parameters’ dynamic noise with the apriori covariance ***Q***.

Instead of constant acceleration model or constant velocity model used for IC-PPP, the dynamic model^31^,^32^ for positions, velocities, and attitudes of INS tightly aided IC-PPP can be expressed as


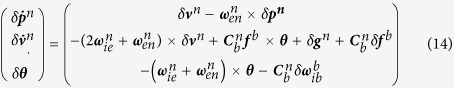


where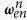 is the rotation angular rate of navigation frame (*n*-frame, North-East-Down) with respect to earth centered earth frame (*e*-frame) projected in *n*-frame; ‘×’ denotes cross-product; 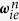 is the earth rotation rate of *e*-frame with respect to inertial frame (*i*-frame) projected in *n*-frame;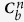 represents the attitude direction cosine matrix for transforming from body frame (*b*-frame, Forward-Right-Down) to *n*-frame; *δ**f***^*b*^, 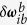, and *δ**g***^*n*^ are offset of accelerometer’s specific force, offset of gyroscope’s angular rate, and gravity offset.

Although there are many hardware errors contained in INS outputs^35^, just biases and scale factors of gyroscopes and accelerometers should be considered for tactical IMU. Usually they can be modelled as 1^st^ Gauss-Markov procedure^36^.


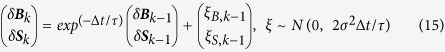


where *τ* is the correlation time; *σ* is the variance of the driving noise (*ξ*) depending on the instability of the IMU biases and scale factors.

Meanwhile, for one multi-GNSS receiver there exit Inter System Biases (ISB) between each pair of two individual GNSS systems due to different frequencies and signal structures adopted by each system. Besides, since the Frequency Division Multiple Access (FDMA) technique is used by GLONASS, it leads to the Inter Frequency Bias (IFB) for every pair of two GLONASS satellites. In order to avoid such errors’ impact on IC-PPP, we estimate 3 independent receiver clock offsets (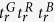) to eliminate the ISBs and 2 + *m* receiver DCBs to remove the IFBs (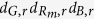). To estimate the receiver clock offset and drift accurately, the model proposed by Brown (1992) is applied, which can be expressed as


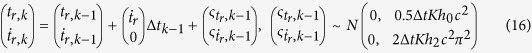


where *h*_0_, *h*_2_ and *K* are the instabilities of the GNSS receiver crystal oscillator and the experiential amplification factor.

The dynamic behavior of the ionosphereic delay variation, receiver DCBs, and the residual of the wet component ZTD can be described by random walk procedure as shown by eqs (5,9 and 10), respectively. Then, the state transfer matrix can be obtained from eqs (5,9,10,14~16). Finally, according to the mathematical algorithm as described above, the closed loop extend Kalman filter^36^ can be employed to update the state parameters and the corresponding variance in the INS tightly aided IC-PPP.

## Results

**Figure 1:**
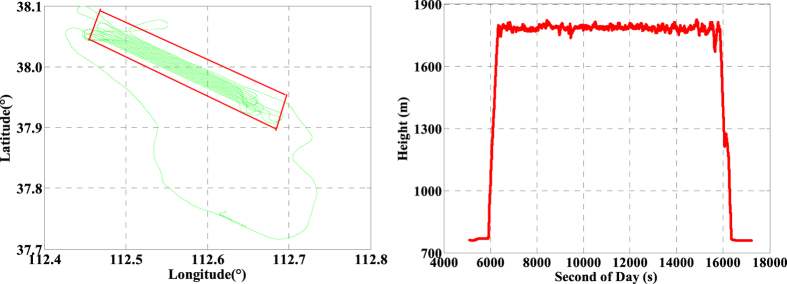
Trajectory (left) and height (right) of the airborne experiment on 25 April, 2015 in Taiyuan, China.

**Figure 2:**
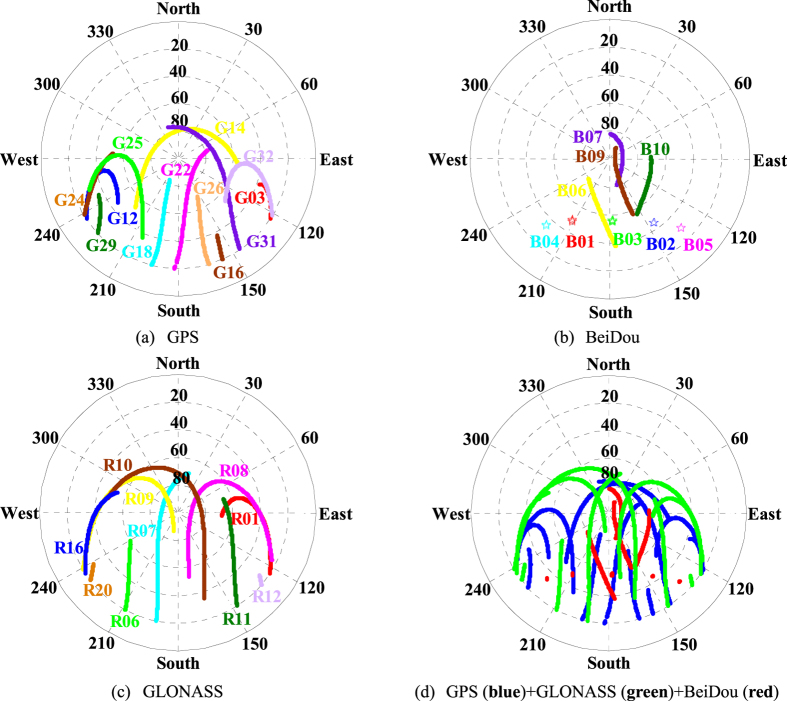
Sky plots (azimuth & elevation) of available multi-constellation GNSS satellites at Taiyuan city, China on 25 April, 2015. The subfigures (**a**) GPS; (**b**) BeiDou; (**c**) GLONASS; (**d**) GPS + GLONASS + BeiDou.

As is well known, the GNSS and INS are usually used to provide the related location information including position, velocity, and orientation for many dynamic applications such as aerial photogrammetry and mobile mapping system. In order to evaluate the performance of the INS tightly aided single- and multi-GNSS IC-PPP in real-time applications, a set of airborne GNSS/INS data from an aerial photogrammetry mission collected in Taiyuan, China on 25 April, 2015 was analyzed. In this experiment, the Trimble R9 multi-GNSS receiver was used to collect GPS, GLONASS, and BeiDou observations (pseudo-range, carrier-phase, and Doppler), and a tactical IMU (POS310) from Wuhan MAP Space Time Navigation Technology Company was used to provide INS data (increments of velocity and angular). The Trimble R9 is a geodetic multi-GNSS receiver with fixing its sampling rate to 1 Hz and the POS310 consists of three fiber-optic gyroscopes and three servo accelerometers with setting the sampling rate to 200 Hz in this test. The whole mission takes about 3.5 hours, and its trajectory is shown in left sub-figure of Fig. 1 with the distances about 41.5 km along North-South direction and 26.9 km in East-West direction, respectively. Shown in the right sub-figure of Fig. 1 is the height of the whole test, and it takes about 2.7 hours in the mission region with the flight height about 1.8 km. The sky plots of available GNSS satellites are displayed in Fig. 2 with the available GPS satellites (2a), the available BeiDou satellites (2b), the available GLONASS satellites (2c), and the three-system available satellites (2d). It is evident that the satellites-users spatial structure in East-West direction is better than that of in North-South direction, which may impact on the positioning accuracy of such two components.

**Figure 3:**
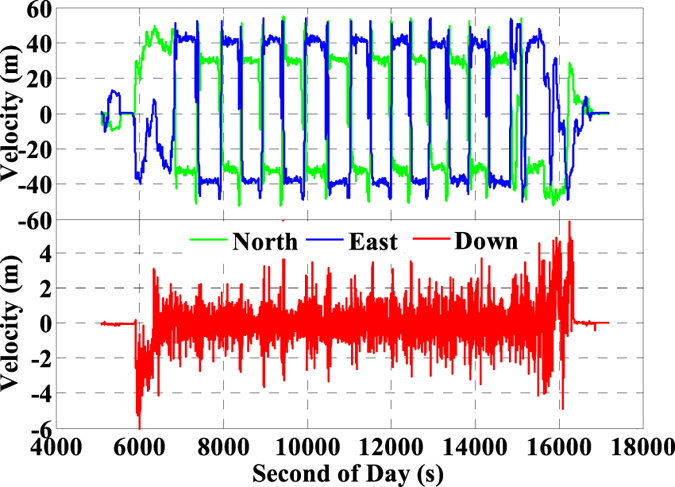
Velocity of the airborne experiment on 25 April, 2015 in Taiyuan, China.

**Figure 4:**
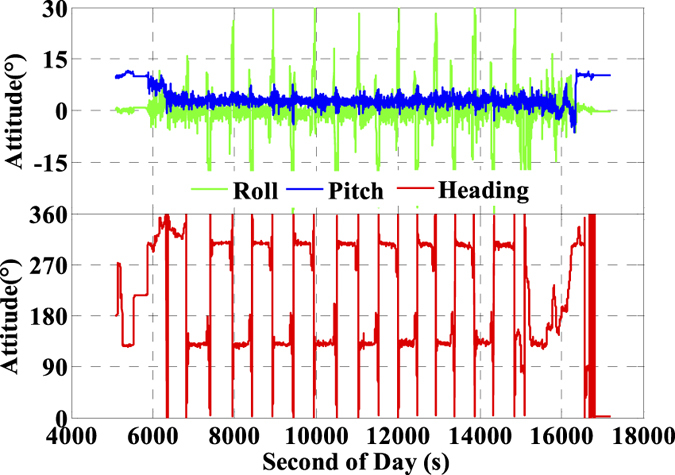
Attitude of the airborne experiment on 25 April, 2015 in Taiyuan, China.

The platform route is arranged into ‘S’ shape with the main directions along West-North to East-South which are shown visibly in Fig. 1. When the airborne-platform goes to the next route from current route periodically, the velocity and the attitude will change uniformly as shown in Figs 3 and 4, respectively. Accordingly, the velocities are about ±55 m/s in horizontal and ±5 m/s in vertical. Meanwhile, the velocity in north and east components (Fig. 3) and attitudes in roll, pitch, and heading directions (Fig. 4) change periodically and significantly due to the ‘S’ route (moved along West-North to East-South). Here, the changes in roll component are the bank angles with the values about ±30 degrees for this mission.

In the data evaluation phase, the GNSS/INS data is processed in the IC-PPP mode and the INS tightly aided IC-PPP mode by using raw pseudo-range and carrier-phase of dual-frequency GNSS data with the ionosphere and receiver DCB constraint. Both of the two stages are processed in kinematic mode with the platform coordinates being estimated at every epoch without any constraints between contiguous epochs. Meanwhile, the ultra-rapid precise satellites orbit (15 minutes interval) and clock (5 minutes interval) products of GPS, GLONASS, and BeiDou provided by GNSS Research Center (GRC), Wuhan University are adopted to weaken the impacts of orbit and clock on positioning accuracy. Besides, the classic error models for GNSS and INS are employed to eliminate the relative errors in PPP and INS tightly aided PPP data process^19^,^20^,^32^. To further close to the real time conditions, all of the GNSS and INS data are processed in forward filter. In additional, the a priori variance values for GNSS raw pseudo-range, carrier-phase, and Doppler are 0.2 m, 0.002 m, and 0.1 m/s, and the process noise values for the wet component of the tropospheric delay, ionosphere delay, receiver DCB, receiver clock offset and drift are 0.01 m/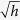, 0.05 m/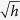, 0.03 m/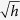, 0.19 m/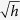 and 0.37 m/s/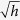. Besides, the IMU related parameter process noises are mainly determined by IMU sensors’ performance. The values for the gyroscope biases and scale factors are 0.5°/*h* and 150 ppm/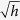, and the values for the accelerometer biases and scale factors are 0.3 m/*s*^2^ and 150 ppm/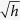.

**Figure 5:**
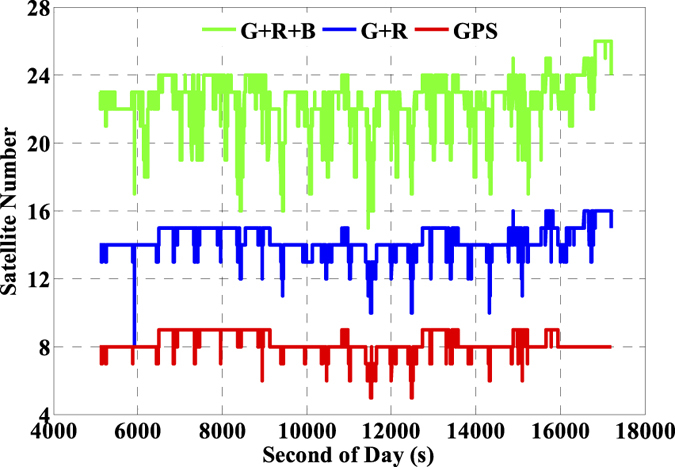
Number of available satellites of GPS (G, red), GPS + GLONASS (G + R, blue), GPS + GLONASS + BeiDou (G + R + B, green) by setting satellite cut-off elevation angles to 10°.

**Figure 6:**
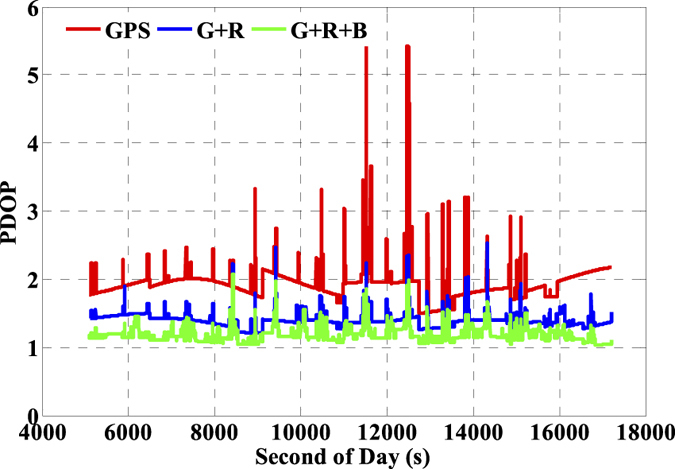
PDOP of GPS (G, red), GPS + GLONASS (G + R, blue), GPS + GLONASS + BeiDou (G + R + B, green) by setting satellite cut-off elevation angles to 10°.

**Figure 7:**
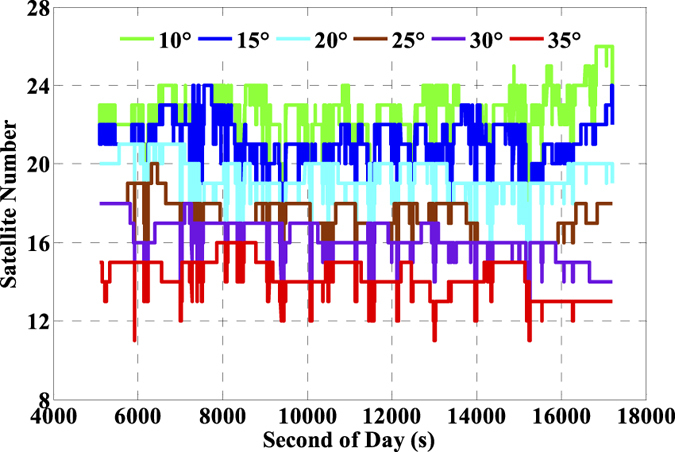
Number of available satellites of G + R + B by setting the cut-off elevation angles from 10° to 35°.

**Figure 8:**
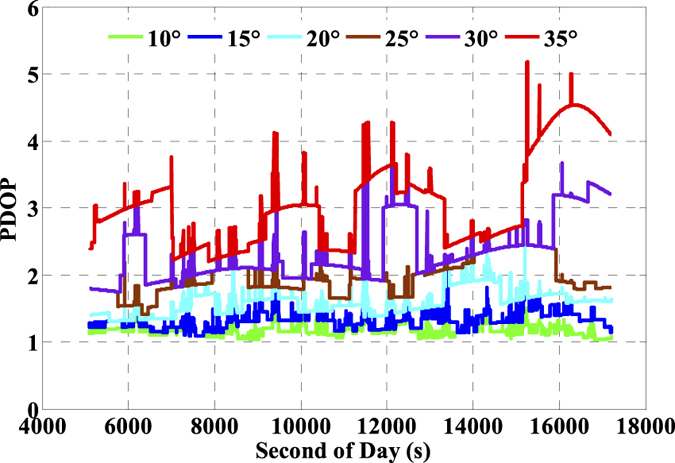
PDOP of G + R + B by setting the cut-off elevation angles from 10° to 35°.

The number of available GNSS satellites and the corresponding PDOP of the single- and multi-GNSS are assessed, and these are vital factors for GNSS precise positioning. The number of available satellites of GPS (G), GPS + GLONASS (G + R), and GPS + GLONASS + BeiDou (G + R + B) with satellite cut-off elevation angle 10° are presented in Fig. 5 and the corresponding PDOP values are depicted in Fig. 6. Accordingly, the averaged number of available satellites and the averaged PDOP are 8.2 and 1.9 while using GPS only. The values are enhanced to 14.3 and 1.4 while using GPS and GLONASS together, and such values are further ameliorated to 22.5 and 1.2, respectively while using the three-system data. It is significant that there are visible improvements in both available satellites and PDOP when multi-GNSS data are used. Besides, we also evaluate the impacts of the number of satellites and PDOP on IC-PPP performance by setting the satellite cut-off elevation angles from 10° to 35° with a stepsize of 5°. According to the results as shown by Figs 7 and 8, it is clear that fewer available satellite number (dropping from 22.5 to 14.2) and larger PDOP (increasing from 1.2 to 3.1) will be obtained along with the satellite cut-off angles increasing. It seems while setting the satellite cut-off elevation angle bigger than 25° the PDOP of G + R + B (Fig. 7) is larger than that of GPS only as shown in Fig. 6, even the number of available satellites is about 2 times larger than that of the GPS only. That is obviously due to the fact that PDOP is not only influenced by the number of available satellites but also constrained by the spatial geometric distribution of these satellites.

**Figure 9:**
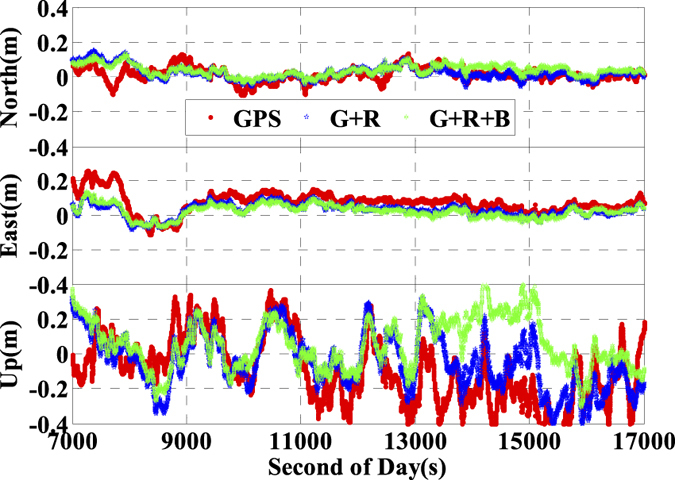
Position differences of ionospheric constraint PPP by using GPS, G + R, and G + R + B comparing with the references values.

**Figure 10:**
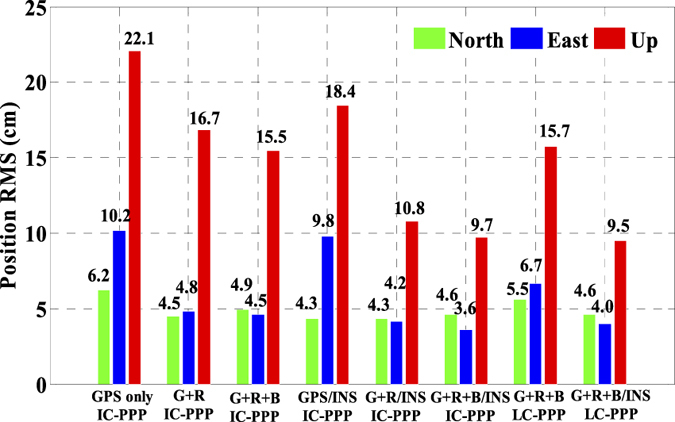
RMS of position differences of IC-PPP and LC-PPP with or without INS aided comparing with the reference values.

In order to validate the performance of the real time kinematic IC-PPP and INS tightly aided IC-PPP, the data is processed in GPS (G), GPS + GLONASS (G + R), and GPS + GLONAS + BeiDou (G + R + B) IC-PPP mode and the corresponding INS aided IC-PPP mode. In the IC-PPP mode, the Global Ionosphere Mapping (GIM) data is adopted as the apriori model for estimating ionospheric delay of each satellite, which is different from the ionospheric delay eliminating method used in the conventional PPP. Meanwhile, the results of GPS RTK (http://www.novatel.com/assets/Documents/Waypoint/Reports/bprocess.pdf) are utilized as the position reference values to validate the final position performance of IC-PPP and INS tightly aided IC-PPP. The position differences between the three IC-PPP schemes and the references values are transformed into local coordinate system (North-East-Up), and the corresponding time series are shown in Fig. 9 by skipping the first 30 minutes solutions. The corresponding statistics indicate that the Root Mean Square (RMS) values of IC-PPP are significantly improved by using G + R and G + R + B data, especially in east and vertical components. According to Fig. 10, the position RMSs of real time kinematic IC-PPP are improved from 6.2 cm, 10.1 cm, and 22.1 cm of GPS only IC-PPP to 4.9 cm, 4.6 cm, and 15.5 cm of G + R + B IC-PPP with an improvement about 20.5%, 54.9%, and 29.8% in North, East, and Up components, respectively. Similarly, there are about 27.9%, 52.6%, and 23.6% improvements when using GPS and GLONASS data together compared to GPS-only solutions. Besides, it seems the solutions of G + R + B IC-PPP are slight worse than these of G + R IC-PPP in north component. It may be caused by the special constellation of BeiDou satellite system at present (5 GEOs over the equator along East-West direction). Generally, the reason for the visible improvements of IC-PPP is that the multi-GNSS can provide more available GNSS satellites and better PDOP.

**Figure 11:**
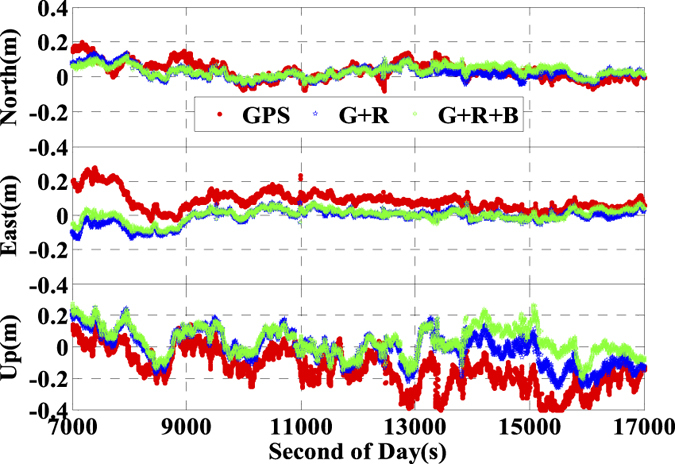
Position differences of INS aided ionospheric constraint PPP by using GPS, G + R, and G + R + B data comparing with the reference values.

**Figure 12:**
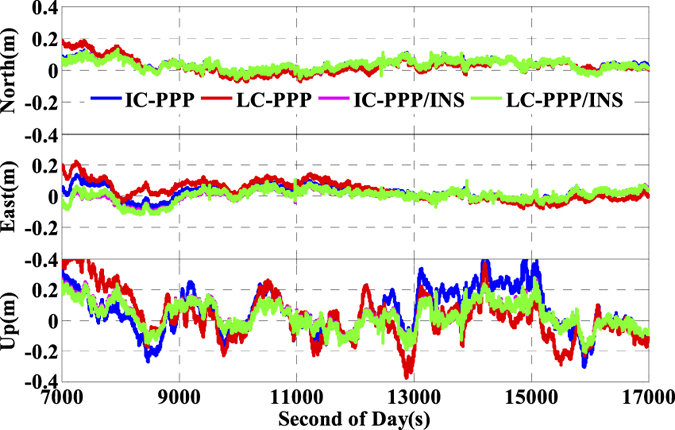
Position differences of IC-PPP and conventional PPP (LC-PPP) with or without INS aided by using G + R + B comparing with the reference values.

Shown in Fig. 11 are the position differences between the INS tightly aided real time kinematic IC-PPP solutions and the references values in North-East-Up system, and the corresponding statistics are listed in Fig. 10. Compared to GNSS IC-PPP solutions, there are evident improvements in terms of standard deviation (STD) and RMS while using INS data to aid IC-PPP. Accordingly, the RMS of the INS tightly aided GPS IC-PPP are 4.3 cm, 9.8 cm, and 18.4 cm with improvements of 42.7%, 3.9%, and 15.9% in North, East, and Up components, respectively with respect to GPS IC-PPP. Analogously, in contrast to G + R + B IC-PPP, there are 6.8%, 21.1%, and 37.3% improvements for the INS aided G + R + B IC-PPP in term of the position RMS (4.3 cm, 3.6 cm, and 9.7 cm) in the three directions. Meanwhile, we also calculate the solutions in conventional PPP (LC-PPP) mode using G + R + B data with and without INS tightly aided. The corresponding results are illustrated in Figs 10 and 12. Similarly, the conventional PPP position RMSs are also enhanced clearly by INS with the average improvement percentages of 17.4%, 40.1%, and 39.7% in North, East, and Up components, respectively. Besides, it seems there is no significant difference between the solutions achieved from the INS tightly aided G + R + B IC-PPP mode and that of the INS tightly aided G + R + B conventional PPP mode (the maximum difference value is less than 0.5 cm). Obviously, INS can refine PPP position accuracy no matter using single- or multi-GNSS data. Concisely, the high rate and high precision INS data are used to predict the current epoch positions, velocities, and attitudes by INS mechanization in the INS tightly aided IC-PPP mode and it provides strong constraints for state parameters between the adjacent epochs. Besides, the high sensitivity of INS data can also weaken the impact of GNSS observation noises in time domain on positioning accuracy.

**Figure 13:**
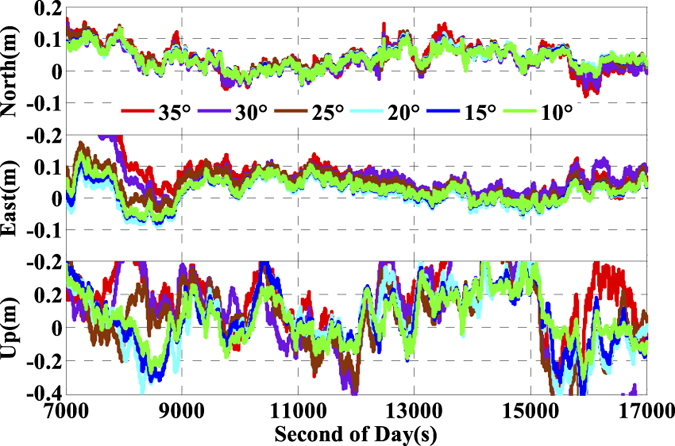
Time series of position offsets between IC-PPP and INS tightly aided IC-PPP using GPS, G + R, and G + R + B data.

**Figure 14:**
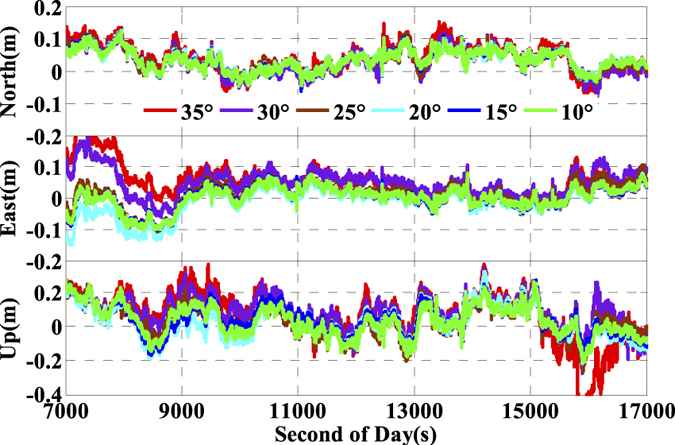
Position differences of INS tightly aided IC-PPP using G + R + B data with setting satellite cut-off elevation angles from 10° to 35°.

**Figure 15:**
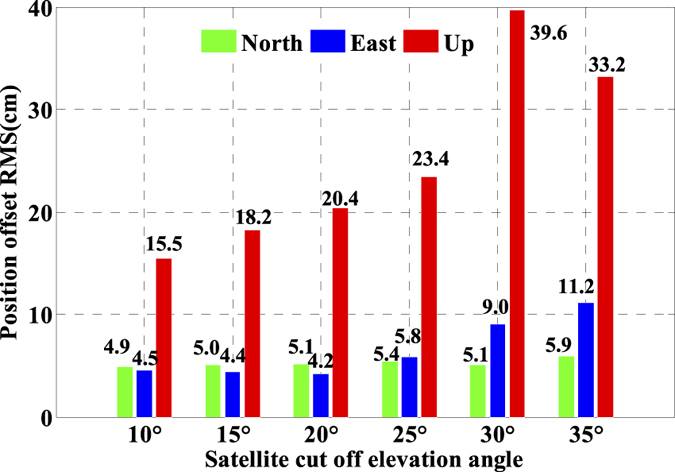
RMS of IC-PPP using G + R + B data with setting satellite cut-off elevation angles from 10° to 35°.

**Figure 16:**
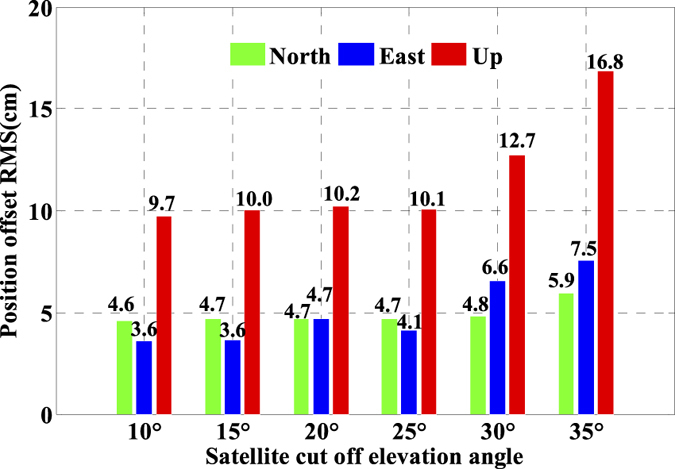
RMS of INS tightly aided IC-PPP using G + R + B data with setting satellite cut-off elevation angles from 10° to 35°.

**Figure 17:**
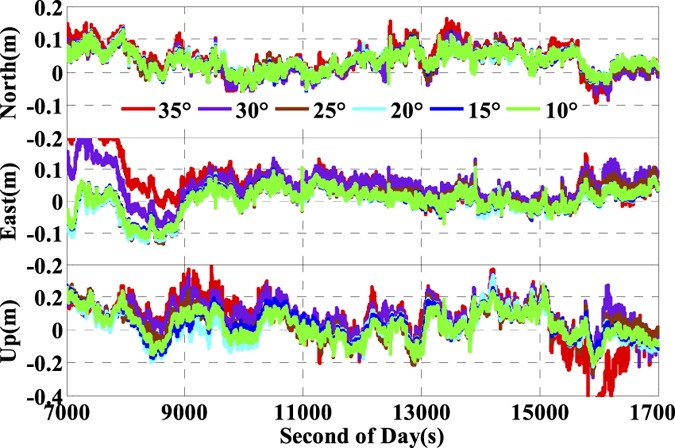
Position differences of INS tightly aided LC-PPP using G + R + B data with setting satellite cut-off elevation angles from 10° to 35°.

**Figure 18:**
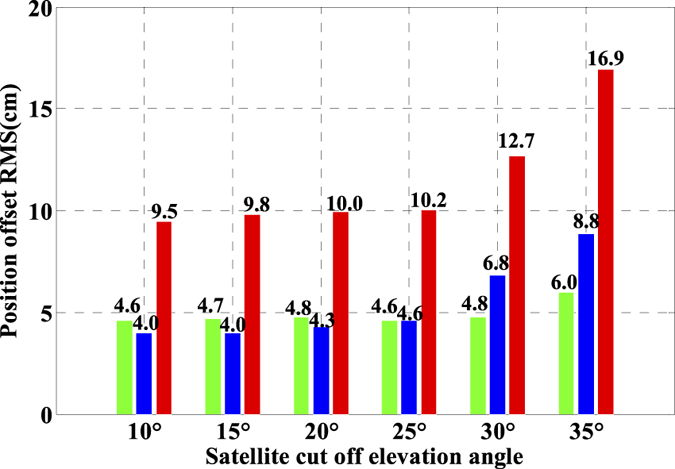
RMS of INS tightly aided LC-PPP using G + R + B data with setting satellite cut-off elevation angles from 10° to 35°.

As is well known, the number of available GPS satellites in dynamic applications may decrease seriously due to the severe user environments, which will degrade GPS position performance. To analyze the multi-GNSS performance in such complex conditions, we did simulations by setting different cut-off elevation angles in the data processing. The varieties of the number of available satellites and PDOP along with different satellite cut-off elevation angles have been assessed in Figs 7 and 8. In order to evaluate its impact on the position accuracy of real time kinematic multi-GNSS PPP, the experimental data are also processed in G + R + B IC-PPP mode and the corresponding INS tightly aided IC-PPP mode under different cut-off elevation angles ranging from 10° to 35° in a stepsize of 5°, and the corresponding position time series are depicted in Figs 13 and 14, respectively. Evidently, we see that the position RMSs of IC-PPP mode (Fig. 15) decrease dramatically from 4.9 cm, 4.5 cm, and 15.5 cm to 5.9 cm, 11.2 cm, and 33.2 cm in North, East, and Up components, respectively when the satellite cut-off elevation angles increase from 10° to 35°, especially in the vertical direction. In contrast, the corresponding solutions of the INS tightly aided IC-PPP exhibit much better accuracy in both RMS and stability. Compared to the RMSs of G + R + B IC-PPP mode, the average improvements of the position RMS using the INS tightly aided IC-PPP are about 6.9%, 19.3%, and 51.0% in North, East, and Up directions. And the improvement in vertical is much more visible than in horizontal. The RMSs of the INS tightly aided G + R + B IC-PPP are dropped from 4.6 cm, 3.6 cm, and 9.7 cm to 5.9 cm, 7.5 cm, and 16.8 cm along with the satellite cut-off elevation angles increasing from 10° to 35° (Fig. 16). What is more, the RMS of the INS aided G + R + B IC-PPP using 35° cut-off elevation angle is better than that of the GPS-only IC-PPP with 10° cut-off elevation angle (see in Fig. 11). Such simulation indicates that it is possible to obtain high accuracy position in complex environment by multi-GNSS (G + R + B) IC-PPP (for example, high buildings in urban city leading to the satellite with low satellite elevation angle cannot be observed). Similar conclusions can also be achieved in the INS tightly aided multi-GNSS conventional PPP mode. The position differences time series and the corresponding statistics are shown in Figs 17 and 18, respectively. Compared to Figs 16 and 18, there is little differences (less 1.0 cm) between the position RMS achieved from the INS tightly aided multi-GNSS IC-PPP and that of the INS tightly aided multi-GNSS LC-PPP.

It is hardly to see the accuracy difference (less than 1.1 cm) in the INS tightly aided G + R + B PPP mode (both IC-PPP and LC-PPP) while setting the satellite cut-off elevation angle under 25°. Comparatively, the position accuracy losses of IC-PPP are about 1.6 cm and 7.9 cm in horizontal and vertical components, respectively. And both the position accuracy of PPP and that of the INS aided PPP are degraded significantly when the cut-off elevation angles are set as 30° and 35°. Theoretically, by setting higher satellite cut-off elevation angle, satellites with low elevation angle will be rejected and fewer GNSS satellites are used in positioning calculation. It can shorten the data processing time with little position accuracy loss as proved in this paper.

**Figure 19:**
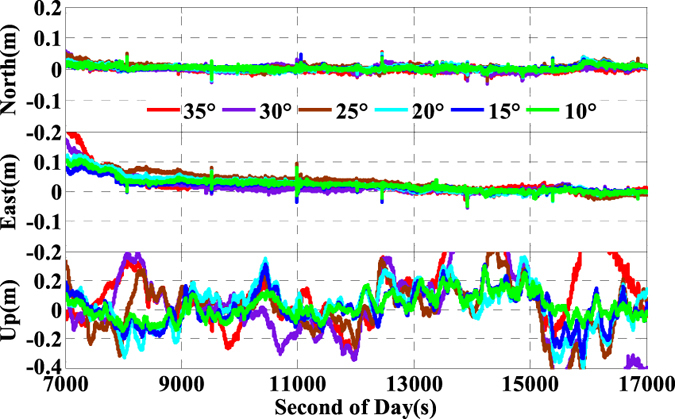
Position offsets time series of between IC-PPP and INS tightly aided IC-PPP using G + R + B data with setting satellite cut-off elevation angles from 10° to 35°.

**Figure 20:**
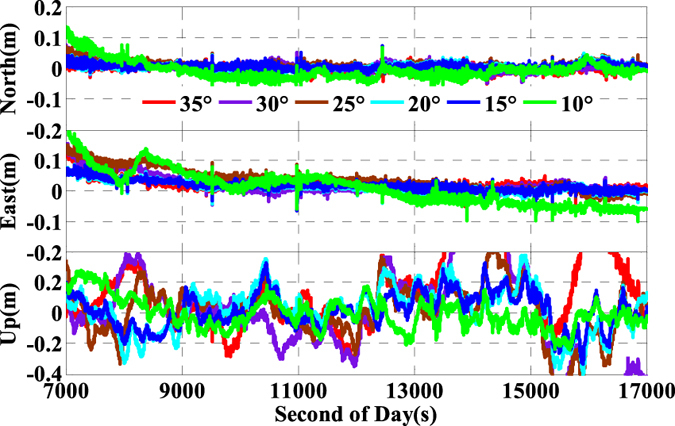
Position offsets time series of between LC-PPP and INS tightly aided LC-PPP using G + R + B data with setting satellite cut-off elevation angles from 10° to 35°.

In order to get the relative relationship between PPP and the INS tightly aided PPP (both IC-PPP and LC-PPP), we made the difference between PPP solutions under different satellite cut-off elevation angles and that of the INS aided PPP, and the corresponding position differences are also transformed into local coordinate system and the corresponding statistics are listed in Table 1. According to statistics, the average position differences RMSs of IC-PPP are 0.9 cm, 3.7 cm and 19.1 cm in North, East, and Up components, respectively, and those of LC-PPP are 1.5 cm, 3.6 cm, and 19.4 cm. Shown in Figs 19 and 20 are the position offsets of IC-PPP and LC-PPP respectively. Accordingly, the discrepancy values in vertical component are enlarged gradually along with the increasing satellite cut-off elevation angle, and such appearance does not show evidently in North and East directions. It means that INS really can enhance the position accuracy of both IC-PPP and LC-PPP, and such enhancements are more obvious in Up component than in horizontal direction. It may be due to the fact that the availability and sensibility of IMU are much better in vertical direction than in other two directions. Besides, as shown in Figs 16 and 18, the position accuracy of the INS tightly aided PPP degrades slightly along with the increasing cut-off angle, which is much different from that of PPP as shown in Fig. 15. It indicates that INS can provide effective improvement for PPP especially when PPP works not well in worse situations (less available satellites number). Such advantage is very important for the real time dynamic precise positioning applications.

Convergence time is also one of the key factors for real-time IC-PPP. According to the research from Bisnath and Gao (2009)^37^, INS is one of the key tools to shorten the PPP convergence time. Gao *et al*. (2015, 2016)^23^,^33^ have evaluated the convergence performances of GPS IC-PPP and multi-GNSS conventional PPP with and/or without INS aided. Here, we assess the convergence performance of the INS tightly aided the multi-GNSS IC-PPP in term of the position accuracy in different time scales.

**Figure 21:**
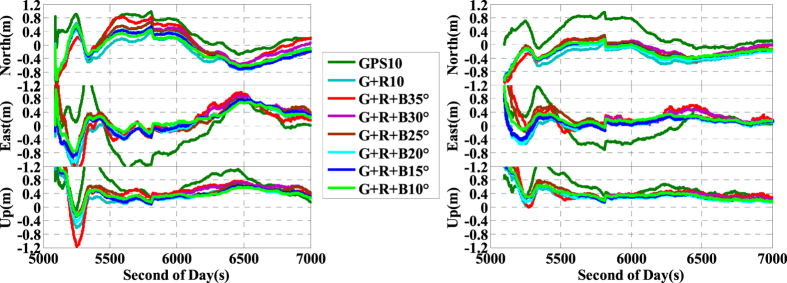
Position differences time series in first 30 minutes calculated by GPS, G + R, G + R + B IC-PPP mode (left) and INS aided IC-PPP mode (right) using different satellite cut-off elevation angles; Here ‘GNSS “X”°’ means use GNSS data with setting the cut-off elevation angle to X°.

Figure 21 shows the convergence performance of IC-PPP and the INS tightly aided IC-PPP with different satellite cut-off elevation angles. According to the left sub-figure in Fig. 21, the convergence of G + R IC-PPP and G + R + B IC-PPP is visibly faster than that of GPS only IC-PPP. Meanwhile, the number of available satellites can also affect the IC-PPP convergence time. The INS tightly aided IC-PPP position time series in the right sub-figure of Fig. 21 expresses a similar impact between its convergence performance and the satellite availability under different cut-off elevation angles. Furthermore, even with satellites cut-off elevation angle of 35°, the convergence of G + R + B IC-PPP and the INS tightly aided IC-PPP (the red line) performs better than GPS only solutions (the deep green line). Besides, comparing the solutions of IC-PPP to those of the INS tightly aided IC-PPP using same GNSS data, it is significant that INS can accelerate the IC-PPP convergence. According to the statistics, when the 30 minutes continuing GNSS observations are used, INS can provide about 24.3%, 44.3%, and 12.9% average position improvements for IC-PPP in North, East, and Up components, respectively. The position RMSs of GPS IC-PPP are improved from 50.8 cm, 69.1 cm, and 76.0 cm to 27.3 cm, 19.4 cm, and 41.0 cm, corresponding to the improvement percentages about 46.1%, 71.9%, and 46.1% in INS tightly aided G + R + B IC-PPP mode with the cut-off elevation angle of 10°. No data are rejected while calculating the position RMS. Similarly, when the 25 minutes continuing GNSS observations are used, the position average improvement percentages are dropped to 23.1%, 39.7%, and 6.6%, the position RMSs are improved from 55.4 cm, 75.0 cm, and 79.7 cm of GPS IC-PPP to 27.9 cm, 20.5 cm, and 44.0 cm of the INS tightly aided G + R + B IC-PPP. Generally, analogous conclusions about the convergence performance of INS aide G + R + B IC-PPP under different cut-off satellite elevation angles can be also obtained. Such characters of the INS aided multi-GNSS IC-PPP can further promote the applications of IC-PPP in real time.

**Figure 22:**
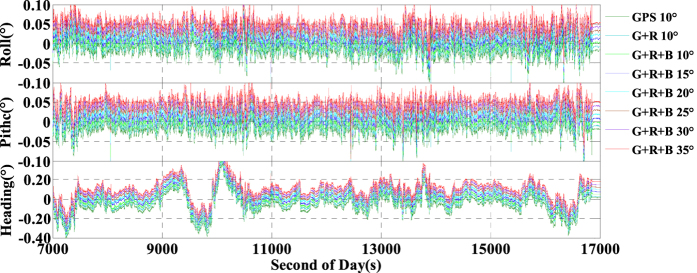
Time series of attitudes calculated by GPS, G + R, G + R + B INS aided IC-PPP mode with setting satellite cut-off elevation angles from 10° to 35°; Here ‘GPS 10°’ means use GPS data and set cut-off elevation angle to 10°; for better view, −0.02°, −0.01°, 0.00°, 0.01°, 0.02°, 0.03°, 0.04° and 0.05° shift are added in the results of GPS 10°, G + R 10°, G + R + B 10°, G + R + B 15°, G + R + B 20°, G + R + B 25°, G + R + B 30°, G + R + B 35° schemes.

For some applications (mapping, navigation, unmanned aerial vehicles etc.), the attitudes determination is also very important. Since no higher precision IMU sensor was used in the test, we just have an assessment about the relative accuracy of the attitudes determination of the INS tightly aided IC-PPP using single- and multi-GNSS data under different satellite cut-off elevation angles. The results are evaluated by comparing with the reference attitudes obtained from the loose integration of RTK and INS. The attitudes accuracy of the RTK/INS (tactical IMU) loosely integration has been evaluated in http://www.novatel.com/assets/Documents/Papers/FSAS.pdf. According to the statistics of attitude differences as depicted in Fig. 22, the RMSs of the attitudes calculated by the eight schemes are extremely close to each other with the maximum discrepancy within ± 0.0006°, and the average RMSs of the attitudes are 0.017°, 0.016°, and 0.104° in roll, pitch, and heading directions, respectively. Besides, the accuracy in heading seems worse than the other two, which may be owing to the worse availability of the gyroscope in heading component.

## Discussion

In order to enhance the performance of real-time PPP, we integrate tightly the multi constellation GNSS IC-PPP with INS to take full advantages of the current available GNSS and INS observations. With a set of GNSS and INS dynamic data from an airborne mission, we evaluate the number of available satellites and PDOP of GPS only, GPS + GLONASS, and GPS + GLONASS + BeiDou under different cut-off elevation angles. The multi-GNSS can significantly increase the number of available satellites and improve the spatial geometry in term of PDOP. Then, the performances of the real-time single- and multi-GNSS IC-PPP and the corresponding INS tightly aided IC-PPP are evaluated. The results indicate that both the multi-GNSS and INS can improve the performance of IC-PPP. In details, the multi-GNSS not only make PPP position time series much more stable, but also provide better positions with average improvements of 20%, 50%, and 30% in North, East, and Up components compared to GPS only solutions. The INS tightly aided IC-PPP mode, can further provide another 6% to 40% position improvements in term of RMS for horizontal and vertical components.

Meanwhile, the position accuracy of both GPS + GLONASS + BeiDou IC-PPP and the corresponding INS tightly aided IC-PPP shows a trend decreasing along with the increasing satellite cut-off elevation angles, especially for IC-PPP mode. The position RMS of the multi-GNSS IC-PPP drops visibly in vertical direction when the cut-off elevation angle increases from 10° up to 35°, but the position accuracy loss in horizontal is hardly to see. In comparison, the position RMS of the INS aided multi-GNSS IC-PPP is about centimeter level even setting the satellite cut-off elevation angle to 35°, and no more than 5 cm position accuracy is lost for all of the three components compared to the best solutions of the INS tightly aided IC-PPP. It means that INS can further improve IC-PPP position accuracy and shorten the data processing time by setting high cut-off elevation angle in real applications. What is more, the INS tightly aided multi-GNSS IC-PPP can significantly improve the convergence speed of GPS only IC-PPP in term of position accuracy. Such advantages of the INS tightly aided IC-PPP as validated in this paper can further promote the applications of PPP in real time. But, the single-/multi-GNSS data and the number of available satellites have little influence on the attitudes resolutions accuracy if there are enough GNSS satellites for positioning.

## Additional Information

**How to cite this article**: Gao, Z. *et al*. Real-time Kinematic Positioning of INS Tightly Aided Multi-GNSS Ionospheric Constrained PPP. *Sci. Rep.* **6**, 30488; doi: 10.1038/srep30488 (2016).

## Figures and Tables

**Table 1:** Relationship between cut-off angles and Position offsets by making difference between multi-GNSS PPP (IC-PPP and LC-PPP) and INS tightly aided PPP.

Cut-off angles	Multi-GNSS IC-PPP (/INS) mode			Multi-GNSS LC-PPP (/INS) mode		
	North (cm)	East (cm)	Up (cm)	North (cm)	East (cm)	Up (cm)
10°	0.8	3.3	8.6	3.0	5.8	10.0
15°	0.8	2.7	11.4	1.0	2.4	11.5
20°	0.9	3.8	14.6	1.1	2.2	14.8
25°	1.2	4.5	18.1	1.5	5.1	18.3
30°	1.0	3.5	34.3	1.2	3.6	34.3
35°	0.8	4.2	27.5	1.1	2.8	27.8
Average	0.9	3.7	19.1	1.5	3.6	19.4
